# Transcriptomal changes and functional annotation of the developing non-human primate choroid plexus

**DOI:** 10.3389/fnins.2015.00082

**Published:** 2015-03-12

**Authors:** C. Joakim Ek, Peter Nathanielsz, Cun Li, Carina Mallard

**Affiliations:** ^1^Department of Physiology, Institute for Neuroscience and Physiology, University of Gothenburg, Sahlgrenska AcademyGothenburg, Sweden; ^2^Department of Obstetrics, Center for Pregnancy and Newborn Research, The University of Texas Health Science CenterSan Antonio, TX, USA

**Keywords:** choroid plexus, development, RNAseq, fetus, primate, non-human, baboon, transcriptome

## Abstract

The choroid plexuses are small organs that protrude into each brain ventricle producing cerebrospinal fluid that constantly bathes the brain. These organs differentiate early in development just after neural closure at a stage when the brain is little vascularized. In recent years the plexus has been shown to have a much more active role in brain development than previously appreciated thereby it can influence both neurogenesis and neural migration by secreting factors into the CSF. However, much of choroid plexus developmental function is still unclear. Most previous studies on this organ have been undertaken in rodents but translation into humans is not straightforward since they have a different timing of brain maturation processes. We have collected choroid plexus from three fetal gestational ages of a non-human primate, the baboon, which has much closer brain development to humans. The transcriptome of the plexuses was determined by next generation sequencing and Ingenuity Pathway Analysis software was used to annotate functions and enrichment of pathways of changes in the transcriptome. The number of unique transcripts decreased with development and the majority of differentially expressed transcripts were down-regulated through development suggesting a more complex and active plexus earlier in fetal development. The functional annotation indicated changes across widespread biological functions in plexus development. In particular we find age-dependent regulation of genes associated with annotation categories: Gene Expression, Development of Cardiovascular System, Nervous System Development and Molecular Transport. Our observations support the idea that the choroid plexus has roles in shaping brain development.

## Introduction

The choroid plexus is a small organ situated in the roof of each brain ventricle. Its main interface is the epithelium that overlays the vascularized core. Its role is often stated as production of cerebrospinal fluid (CSF), which acts to flush out waste products of the central nervous system (CNS), a role that is often referred to as the sink effect. However, there is an increasing awareness of the importance of this organ and the CSF in relation to brain development and normal brain function throughout the life-course. The CSF influences both neurogenesis and neuronal migration having a role during development, in the adult, in aging and after brain trauma (Miyan et al., 2003; Redzic et al., 2005; Sawamoto et al., 2006; Falcao et al., 2012; Zappaterra and Lehtinen, 2012; Lehtinen et al., 2013). In addition, the choroid plexus is today thought to play a role as an immune sensing organ for the brain as well as serving as a point of entry for immune cells into the CNS (Ransohoff and Engelhardt, 2012). CSF secretion by the plexuses is impressive. It turns over the total CSF volume (about 150 mL in a human adult) 3–4 times each day. In addition, the plexuses secrete a wide range of signaling molecules (Lehtinen et al., 2013).

The role of the choroid plexus in development may be of particular importance since it differentiates very early during fetal life, just after neural tube closure, at a time of early cortical vascularization. In the mouse, the rombencephalic choroid plexuses start to differentiate at around E12 and in humans it occurs at around 7 weeks gestation with the telencephalic choroid plexus (tCP) and diencephalic soon after (Jacobsen et al., 1982; Dziegielewska et al., 2001). The size of the plexuses is very much larger in the embryo/fetus in relation to brain size than in the adult (Johanson, 1995). Soon after the plexuses are formed they occupy almost the whole ventricular space at a stage when the cortex is just a thin layer of cells. One important anatomical relationship is the close opposition of the lateral choroid plexus to the ventricular surface of the developing brain where most neurogenesis occurs, suggesting that factors secreted by this plexus are in a position to influence brain development (Falcao et al., 2012). CSF in developing animals has a much higher protein concentration than in adults (Dziegielewska et al., 2000), a difference that was previously thought to be a consequence of an open blood-CSF barrier in early life. However, there is now significant evidence that this barrier closes early in fetal life (Ek et al., 2003; Liddelow et al., 2013). However, the exact contribution of the choroid plexus to the protein composition of fetal CSF is still not clear, as it is able to contribute to both secretion and absorption of a variety of molecules thereby regulating the CSF environment.

With the availability of powerful new molecular techniques, some of the early signaling systems, such as the bone morphogenetic proteins and the sonic hedgehog signaling, that control plexus differentiation have now been proposed (Lehtinen et al., 2013). These authors highlight that the choroid plexus is one of the most under-studied organs in the brain suggesting that new roles for the plexus remain to be discovered. Nearly all the studies of development and function of the choroid plexus have been undertaken in altricial rodent models (Kratzer et al., 2013; Liddelow et al., 2013; Saunder et al., 2013), which have a shorter prenatal development than more precocial species such as humans and non-human primates. There are key differences in the developmental trajectory and regulation between precocial and altricial species (Rabadan-Diehl and Nathanielsz, 2013). Thus, studies in precocial species are needed to remove this barrier to our understanding. To obtain information that will help address this need, we present here information on gene expression in the lateral choroid plexus tissue at three developmental stages in the baboon fetus which shows much closer development to humans. Baboons present one of the best opportunities for comparative studies of maturation of neural structures in fetal life with human fetuses. For example, they show features such as cerebral gyrification index in development similar to humans (Rogers et al., 2010). Our study of choroid plexus development was conducted during the second half of gestation when most of the cortical expansion is occurring in primates (Kroenke et al., 2007). We analyzed the full transcriptome identified using Next Generation Sequencing and used the Ingenuity knowledge base to annotate functions, enrichment of pathways and to predict regulatory networks of changes in the transcriptome.

## Materials and methods

### Care of animals

Fourteen female baboons (*Papio hamadryas*) from the Southwest National Primate Research Center (San Antonio, TX, USA), were recruited for this study and maintained in group housing. All procedures were approved by the Texas Biomedical Research Institute Institutional Animal Care and Use Committee and conducted in AAALAC-approved facilities. The caging system allows control and monitoring of food intake while still maintaining female baboons in group housing, thereby permitting normal social and physical activity (Schlabritz-Loutsevitch et al., 2004). Feeding and management have previously been described in detail (Li et al., 2013). Cesarean sections were performed between 0800 and 1000 h at 90, 120, and 165 days of gestation (term 184 days) under general anesthesia using techniques previously reported in detail (Schlabritz-Loutsevitch et al., 2007). Food was withdrawn for 16 h before surgery. Post-operative analgesia was provided with buprenorphine hydrochloride 0.015 mg/kg per day (Hospira, Inc., Lake Forest, IL, USA) for three post-operative days. Fetal tCP was collected and flash frozen in liquid nitrogen at 90 days gestation (3 males/1 female), 120 (2 males/2 females) and 165 day (3 males/3 females) of gestation, corresponding to 0.5, 0.7, and 0.9 gestation.

### RNA preparation and Illumina next generation sequencing

Frozen tissues were homogenized with a mechanical homogenizer in 400 μL RNase free PBS. RNA was extracted with an RNAeasy mini-kit (Qiagen) according to manufacturer's recommendations along with DNA digestion. RNA quality was analyzed by Biorad Experion electrophoresis. RNA sequencing was performed at the Genomics Core Facility at University of Gothenburg. 1 μg of high quality total RNA (RIN > 8) was used for library preparation. Libraries were created using the TruSeq™ RNA Sample Preparation v2 kit, according to the manufacturer's protocol (TruSeq™ RNA Sample Preparation v2 Guide). The library was subjected to 50 bp single end read cycles of sequencing on an Illumina HiScan SQ as per manufacturer protocol.

### Analysis of sequencing data

Quality assessment of the sequence reads was performed by generating QC statistics with FastQC (http://www.bioinformatics.bbsrc.ac.uk/projects/fastqc). RNA-seq reads were mapped to the current baboon genome assembly, Pham_1.01 (https://www.hgsc.bcm.edu/content/baboon-genome-project) using Tophat (Trapnell et al., 2009). Cufflinks (Trapnell et al., 2012) was used for transcript assembly of individual samples. All assemblies were merged to create a reference transcript, which was used to calculate gene counts with HTSeq (http://www-huber.embl.de/users/anders/HTSeq/doc/overview.html). One of the replicates from GD90 was removed from the analysis since it was found to be extremely different from the other replicates. DESeq (Anders and Huber, 2010) was then used to find differentially expressed genes. Annotation of genes was done through blasting (Altschul et al., 1990) each transcript against the Uniprot database (UniProt Consortium, 2012). Transcripts with a *p*-value of less than 0.05 after false discovery rate correction were considered to be significantly differentially expressed. Proteins with human homologs were used as input to for analysis in Ingenuity Pathway Analysis software (IPA), a program that serves for the interpretation of large scale transcriptome data across species, with a fold change cut-off of 2.0 and *p* < 0.05 across all analysis. This program creates these analyses using the Ingenuity knowledge base, a huge database containing millions of individual interactions between genes and proteins. Analysis using IPA was conducted between June-September 2014 (Ingenuity version 18841524; www.ingenuity.com). A core analysis was created in IPA using standard settings with duplicates resolved with average fold changes.

## Results

### Whole genome expression profiling

We used next generation sequencing to analyze fetal developmental changes in the tCP transcriptome. In total 75,760 transcripts were detected (≥1 transcript) across all tCP samples with 70,821 transcripts at GD90, 71,625 at GD120 and 72,651 at GD165.

**Figure 1:**
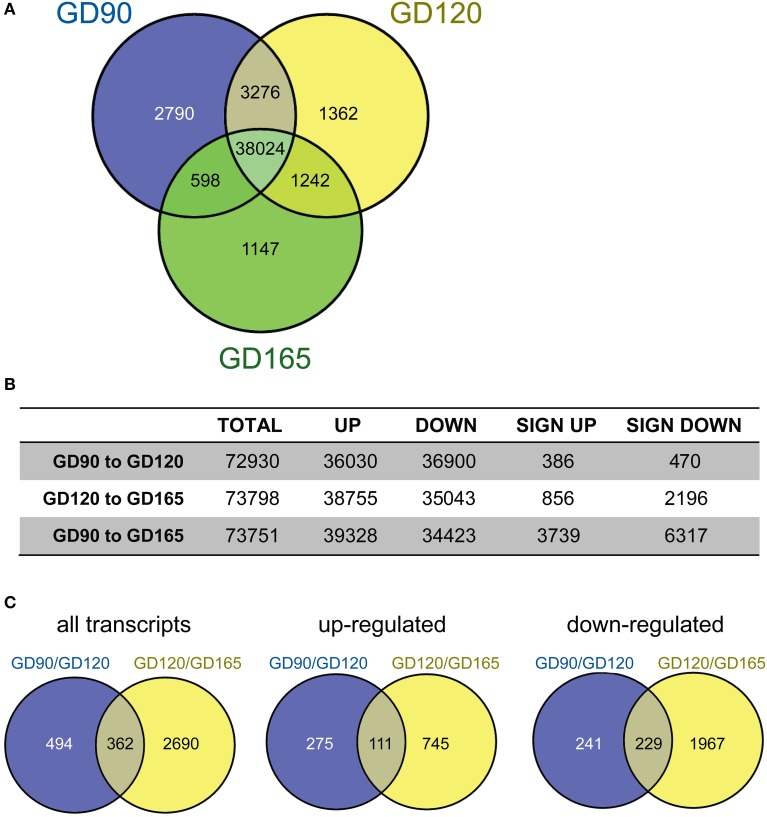
**(A)** Venn-diagram showing the number of transcripts present at gestational day (GD) 90 (blue), 120 (yellow), and 165 (green) in the fetal baboon choroid plexus. A transcript was considered present when the majority of animal at each age had >1 transcript and the median number of transcripts was >10. The majority of transcripts were present at all ages (78%). The number of unique transcripts decreased with developmental age with 2790 at GD90, 1362 at GD120 and 1147 at GD165. **(B)** Pairwise comparisons of the number of up/down regulated transcripts (non-significant) as well as significantly regulated transcripts between the three fetal ages. The total number of significantly regulated transcripts was less between GD90 and GD120 (856) than between GD120 and GD165 (3052) and greatest between GD90 and GD165 (10056). **(C)** Number of significantly regulated transcripts that were in common between the two pairwise comparisons GD90/GD120 and GD120/GD165 (left) as well as up-(middle) and down-regulated (right) transcripts.

In Figure 1A the number of transcripts in common and unique in the tCP at GD90, GD120 and GD165, is depicted by a Venn-diagram. For this illustration, a transcript was considered present when the majority of animals had >0 transcripts and the median number of transcripts (read counts) was ≥10 at each gestational age. This shows that the majority of transcripts (78.4%) were common across all gestational ages. The number of transcripts unique (i.e., only present within a single group) at GD90 was 2790 transcripts (5.8% of total within this group), at GD120 was 1362 (2.8%) and at GD165 was 1147 (2.4%). The number of transcripts unique to the GD90/GD120 groups was 3276 (6.7% of total), in the GD120/165 groups it was 1242 (2.6%) and in the GD90/165 groups it was 598 (1.2%). Pairwise analysis of differentially expressed transcripts between gestational ages was carried out for the input into IPA. As seen in Figure 1B, the total number of differentially expressed transcripts was greater between GD120 and GD165 (3052 or 4.1% of total) than between GD90 and GD120 (856 or 1.1%) and overall between GD90 and GD165 more than 10,000 transcripts (13.6%) were differentially expressed. There was also a greater number of significantly down-regulated over up-regulated transcripts both from GD90 to GD120 (45% up, 55% down) and from GD120 to GD165 (28% up, 72% down). The number of differentially expressed transcripts in common between the pairwise comparisons is presented in Figure 1C. About 29% of transcripts that were significantly up-regulated between GD90 and GD120 were also significantly up-regulated between GD120 and GD165 and about 49% of transcripts significantly down-regulated between GD90 and GD120 were also down-regulated between GD120 and GD165. Only 20 transcripts in total were significantly regulated in opposite directions (up/down or down/up) between the two pairwise comparisons.

### Functional annotation: GD90/GD120 and GD120/GD165

Ingenuity pathway analysis software was used for the functional annotation of differentially expressed genes. This uses the Ingenuity knowledge base to functionally annotate genes and to predict the biological functions of these changes (downstream effect analysis). The tendency (direction) of a biological function can in this way be predicted (activation z-score; >2.0 or <-2 is significantly predictive). These functions are classified under three main groups: Physiological System Development and Function, Molecular and Cellular Mechanisms and Diseases & Disorders. The functional annotation showed that the top five functional categories were similar between the pairwise comparisons of GD90/GD120 and GD120/GD165. For Physiological Systems the annotations were all related to tissue development or survival with Embryonic development, Organ development and Organismal development common in the top five for the two pairwise comparisons. Similarly, for Molecular and Cellular Functions 3/5 groupings were in common (Cellular movement, Cellular Assembly and Organization, and Cell Morphology). It should also be mentioned that 4/5 top Diseases and Disorders in the functional annotation were identical in the two pairwise analysis (Cancer, Organismal Injury and Abnormalities, Reproductive System Disease and Gastrointestinal Disease). Furthermore, a more comprehensive comparison analysis of the pairwise functional annotation was also made between the GD90/GD120 and GD120/GD165. In Table 1 are the top 25 activation z-scores presented for all biological functions. Only biological functions where an activation z-score could be calculated for both pairwise comparisons have been included. Twenty-two out of top 25 Disease/Biofunctions showed the same directionality and the three opposing activation scores were all found in the lower half of the Table with two of these being closely related (Proliferation of cells and Quantity of cells). The most notable difference between age comparisons was that Migration of Cells had a positive activation score for the GD90/GD120 comparison (+1.15; non-significant), whereas a negative activation score was found for the GD120/GD165 comparison (−4.06; significant). Although many of these predicted effects such as disease related changes may not be directly relevant for choroid plexus developmental function, it indicates that the functional annotations of the transcriptional changes throughout fetal tCP development are quite uniform. For these reasons further analysis was focused on differential changes between GD90 and GD165 and annotations to diseases and disorders were not further considered.

**Table 1:**
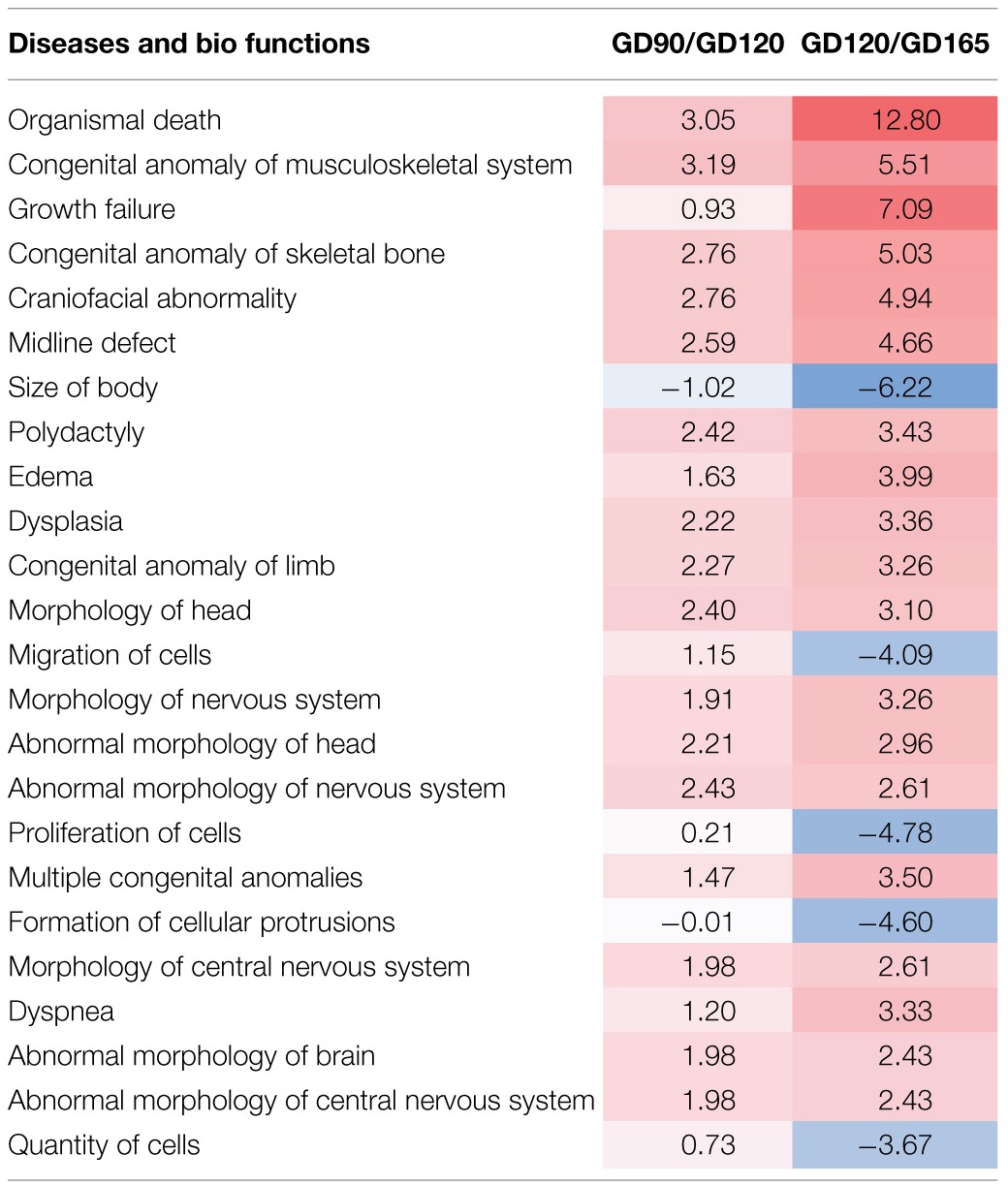
**Comparison analy1sis of functional annotation for the pairwise GD90/GD120 and GD120/GD165 comparisons**.

*Values are activation z-scores of Diseases and Biofunctions in order of highest scoring with values color indexed red for positive scores and blue for negative scores. All but three functions show the same directionality in z-score (most notably exception being Migration of cells). Many of these functions can be expected to have little relevance for choroid plexus function but still suggests that the transcriptomal changes in plexus are relatively consistent. Note that the comparison GD120/GD165 consistently show higher/lower activation scores than the GD90/GD120*.

### Analysis of transcriptional changes between GD90 and GD165

#### Functional annotation

All significant biological functions of highly regulated categories are presented in Tables 2A,B sorted after *p*-values with 19 categories for Molecular and Cellular Mechanisms and with 20 categories for Physiological Systems and Development. A complete list with all subcategories is presented in Supplementary Table 1 for all annotated functions with a significant activation z-score. Based on the potential significance specifically for choroid plexus function and development we selected four highly regulated categories (Gene Expression; Development of Cardiovascular System; Nervous System Development and Molecular Transport) for further detailed analysis. These functions were all significantly enriched in the functional annotation (Tables 2A,B).

**Table 2A:** **Functional annotation under Physiological System Development and Function of genes significantly changed between GD90 and GD165**.

**Category**	***p*-values**
Organismal survival	1.50E-14–1.23E-03
Cardiovascular system development and function	1.50E-14–1.25E-03
Embryonic development	3.49E-14–1.19E-03
Organismal development	2.87E-11–8.44E-04
Nervous system development and function	2.87E-11–1.23E-03
Tissue development	4.04E-10–1.46E-05
Organ development	5.25E-10–1.23E-03
Organ morphology	4.23E-09–1.23E-03
Respiratory system development and function	6.01E-08–1.11E-03
Tissue morphology	6.01E-08–1.23E-03
Visual system development and function	6.01E-08–1.09E-03
Connective tissue development and function	3.03E-07–1.19E-03
Skeletal and muscular system development and function	3.05E-07–3.05E-07
Digestive system development and function	9.14E-07–1.25E-03
Behavior	3.76E-06–6.16E-04
Hair and skin development and function	1.00E-04–9.29E-04
Renal and urological system development and function	1.92E-04–1.2E-03
Reproductive system development and function	4.02E-04–4.02E-04
Lymphoid tissue structure and development	4.02E-04–1.11E-03
Hematological system development and function	4.38E-04–9.29E-04

*The p-value is the probability that the gene changes are related to a particular function just by chance. These are given as ranges since there are several subcategories under each of these top categories*.

**Table 2B:** **Functional annotation under Molecular and Cellular Functions of genes significantly changed between GD90 and GD165**.

**Category**	***p*-values**
Cellular assembly and organization	2.64E-18–9.48E-04
Cellular function and maintenance	2.64E-18–9.48E-04
Cellular growth and proliferation	7.27E-14–1.22E-03
Cell morphology	1.33E-12–1.16E-03
Cellular movement	2.87E-11–1.2E-03
Cellular development	2.83E-10–5.57E-04
Cell death and survival	4.06E-07–1.11E-03
Post-translational modification	6.93E-07–1.07E-03
Cell cycle	6.95E-06–1.11E-03
Cell-to-cell signaling and interaction	2.57E-05–6.44E-04
Gene expression	9.51E-05–2.8E-04
Molecular transport	9.51E-05–1.07E-03
DNA replication, recombination, and repair	1.9E-04–1.07E-03
Amino acid metabolism	2.05E-04–2.05E-04
Small molecule biochemistry	2.52E-04–4.29E-04
Cell signaling	4.53E-04–4.53E-04
Carbohydrate metabolism	9.29E-04–9.29E-04
Cellular compromise	9.29E-04–9.29E-04
Protein synthesis	9.34E-04–1.41E-03

*The p-value is the probability that the gene changes are related to a particular function just by chance. These are given as ranges since there are several subcategories under each of these top categories*.

##### Gene expression

The majority of significantly regulated transcripts were down-regulated in our dataset between GD90 and GD165 (see Figure 1). Table 3A lists all sub-categorized functions for gene expression functions. All genes annotated to Transcription, Transcription of RNA/DNA, Expression of RNA/DNA and Transactivation showed overall significant negative activation scores (−2.84 to −5.22) predicting inhibition in all these functions. It should be noted that there is a very high level of overlap between the genes associated with the first five of these functions (>90%) but also Transactivation show substantial overlap of genes with Transcription (about 75%). We have presented the 20 most regulated genes (based on fold change) predicting decrease in Transactivation (elements working in trans to regulate transcription), the category which showed the most negative activation score (−5.22), see Table 3B. In total 98 gene changes predicted a decrease in Transactivation and 38 predicted an increase out of a total of 149 genes annotated to this function. The number of findings of each predicted direction of function (increase or decrease) for each gene is also presented in this Table as a measure of robustness for the prediction. In Table 3B, and in other similar Tables showing top regulated genes, we have only included genes for which there are at least 2 findings in the Ingenuity knowledge base predicting consistent change in function (checked for each gene in all lists).

**Table 3A:** **Subcategory functions for genes annotated to gene expression along with predictions for activation state**.

**Functions annotation**	**z-score**	**Prediction**	**#**
Transcription	−4.00	Decreased	428
Transcription of DNA	−2.84	Decreased	319
Transcription of RNA	−3.80	Decreased	419
Expression of DNA	−3.36	Decreased	337
Expression of RNA	−4.15	Decreased	464
Transactivation	−5.22	Decreased	149

*#, Number of annotated genes to function*.

**Table 3B:** **The 20 most genes regulated genes predicting a decrease in Transactivation between GD90 and GD165**.

**Gene ID**	**Fold change**	**Findings**	**#**
*CREBBP*	−5.30	Increases	(30)
*NCOA2*	−4.53	Increases	(32)
*EP300*	−4.32	Increases	(38)
*MYBL2*	−3.99	Increases	(2)
*SMARCC1*	−3.96	Increases	(2)
*TRRAP*	−3.89	Increases	(4)
*NFE2*	−3.74	Increases	(2)
*CCNT1*	−3.72	Increases	(4)
*BMPR1B*	−3.46	Increases	(2)
*MITF*	−3.43	Increases	(2)
*GLI2*	−3.35	Increases	(2)
*TWIST1*	−3.25	Increases	(2)
*NCOA1*	−3.23	Increases	(35)
*MED1*	−3.22	Increases	(4)
*UBR5*	−2.97	Increases	(2)
*NOTCH1*	−2.95	Increases	(7)
*E2F1*	−2.86	Increases	(6)
*PRKG1*	−2.83	Increases	(2)
*NR3C1*	−2.75	Increases	(38)
*IRF7*	+2.74	Decreases	(2)

*In total 98 gene changes predicted a decrease in transactivation and 38 predicted an increase out of total 149 genes in data set. Number in brackets is number of findings that the prediction is based upon*.

##### Cardiovascular system development and function

Since the choroid plexus is a highly vascularized tissue we were particularity interested in genes related to cardiovascular function. In the functional annotation for System Biology this category scored the second most significant *p*-value (Table 2A). Functions categorized to Cardiovascular Development and Function are presented in Table 4A with predicted inhibition in Development of blood vessels, Angiogenesis, Morphogenesis, Vasculogenesis, and Formation of blood vessels whereas the Morphology of Cardiovascular System was predicted to increase, Table 4A. There is a considerable overlap of genes annotated to these functions so we have only presented genes associated with Vasculogenesis which had the most significant z-score. From a total of 245 genes of genes annotated to Vasculogenesis, 107 were predictive of decrease in function whereas 67 predicted increase. In Table 4B we have presented the 20 most regulated genes predicting decrease in Vasculogenesis.

**Table 4A:** **Subcategory functions for genes annotated to Cardiovascular System along with predictions for activation state**.

**Functions annotation**	**z-score**	**Prediction**	**#**
Development of cardiovascular system	−2.58	Decreased	328
Angiogenesis	−2.39	Decreased	234
Morphogenesis of cardiovascular system	−3.01	Decreased	72
Development of blood vessel	−2.46	Decreased	268
Vasculogenesis	−3.15	Decreased	245
Formation of blood vessel	−2.15	Decreased	33
Morphology of cardiovascular system	3.06	Increased	224

*#, Number of annotated genes to function*.

**Table 4B:** **The 20 most regulated genes predicting a decrease in Vasculogenesis between GD90 and GD165**.

**Gene ID**	**Fold change**	**Findings**	**#**
*CDH13*	−52.11	Increases	(5)
*NPPC*	43.88	Decreases	(2)
*SLC8A1*	−11.58	Increases	(2)
*EDIL3*	−8.34	Increases	(2)
*SPP1*	−8.13	Increases	(3)
*MEOX2*	−6.87	Increases	(2)
*VASH2*	−6.87	Increases	(2)
*SEMA5A*	−6.01	Increases	(2)
*SLIT2*	−5.99	Increases	(2)
*COL4A3*	5.50	Decreases	(3)
*FGF13*	−5.19	Increases	(6)
*ANPEP*	−4.36	Increases	(2)
*ETS1*	−4.23	Increases	(3)
*MDK*	−4.15	Increases	(3)
*ANGPT1*	−4.11	Increases	(20)
*EFNB2*	−4.04	Increases	(24)
*PIK3C2A*	−4.04	Increases	(2)
*CDH5*	−4.02	Increases	(22)
*MTOR*	−3.95	Increases	(3)
*THBS2*	3.75	Decreases	(3)

*From a total of 245 annotated genes, 107 are predictive of inhibition of function whereas 67 predict activation. Number in brackets is number of findings that the prediction is based upon*.

##### Nervous system development and function

The tCP is situated in close proximity to the subventricular zone, an area important for neurogenesis. In the downstream effect analysis of System Biology the category Nervous System Development scored the 5th most significant *p*-value (Table 2A). Subcategory functions are presented in Table 5A along with activation z-scores. All functions scored negative activation scores (range −2.01 to −3.23) except those related to Morphology of Central Nervous System, which had positive activation scores (+3.13 to +4.01). We have further presented genes for Formation of neurites and Morphology of CNS as these two had the most negative and positive activation z-scores (Tables 5B,C).

**Table 5A:** **Functions annotation for Nervous System Development along with predictions for activation state (z-score)**.

**Functions annotation**	**z-score**	**Prediction**	**#**
Development of brain	−2.01	Decreased	157
Migration of neurons	−2.66	Decreased	89
Proliferation of neuronal cells	−2.75	Decreased	174
Extension of neuritis	−2.20	Decreased	58
Guidance of axons	−2.59	Decreased	55
Formation of neuritis	−3.23	Decreased	59
Long-term potentiation of brain	−2.04	Decreased	43
Long-term potentiation, hippocampus	−2.36	Decreased	38
Long-term potentiation, cerebral cortex	−2.38	Decreased	39
Plasticity of synapse	−2.72	Decreased	36
Morphology of CNS	4.06	Increased	162
Morphology of brain	4.06	Increased	148
Morphology of forebrain	3.13	Increased	75
Morphology of telencephalon	3.13	Increased	60

*#, Number of annotated genes to function*.

**Table 5B:** **Genes predicting an inhibition of Formation of neurites between GD90 and GD165**.

**Gene ID**	**Fold change**	**Finding**	**#**
*MAP1B*	−7.15	Increases	(6)
*ROBO1*	−4.52	Increases	(2)
*CDH2*	−3.46	Increases	(2)
*EFNB1*	−3.18	Increases	(3)
*SLC9A1*	−2.48	Increases	(4)
*SKIL*	−2.42	Increases	(2)
*DIXDC1*	−2.40	Increases	(2)
*SNCA*	−2.32	Increases	(3)
*SEMA4D*	−2.20	Increases	(3)

*In total 20 genes predicted an increase in morphology of CNS with 5 predicting a decrease from total of 59 genes. Number in brackets is the number of findings in in ingenuity knowledge base predicting the functional outcome*.

**Table 5C:** **All genes predicting an increase in morphology of CNS between GD90 and GD165**.

**Gene ID**	**Fold change**	**Finding**	**#**
*FEZF1*	−16.15	Decreases	(2)
*MAP1B*	−7.15	Decreases	(3)
*DCC*	−5.54	Decreases	(2)
*ZNF423*	−4.90	Decreases	(2)
*GLI3*	−4.07	Decreases	(2)
*NFIB*	−3.61	Decreases	(2)
*NDST1*	−3.38	Decreases	(2)
*EPHA4*	−3.00	Decreases	(3)
*NR1H3*	−2.49	Decreases	(2)
*NFIA*	−2.42	Decreases	(3)
*LHX2*	−2.42	Decreases	(3)
*CCND2*	−2.27	Decreases	(2)

*In total 17 genes predicted an increase in morphology of CNS with none predicting an increase from total of 162 genes. Number in brackets is number of findings that the prediction is based upon*.

##### Transport of molecule

Since the choroid plexuses is one of the interfaces between blood and the CNS, is the main source of CSF and is known to be rich in transporter proteins we have further explored genes associated to Transport of Molecule. This subcategory scored an activation z-score of −1.81 (non-significant). The top 25 regulated up/down genes between GD90 and GD165 are presented in Table 6.

**Table 6:** **Top 25 changed genes annotated to Transport of Molecule based on fold change (both up/down-regulated genes)**.

**Gene ID**	**Prediction**	**FC**	**Findings**	**#**
*KCNJ5*	Increased	−51.21	Decreases	(2)
*KCNJ1*	Decreased	−39.25	Increases	(3)
*SLCO1C1*	Increased	+27.45	Increases	(10)
*CFTR*	Decreased	−14.00	Increases	(30)
*CP*	Increased	+10.78	Increases	(7)
*IL6*	Increased	+8.97	Increases	(10)
*SLC1A7*	Decreased	−7.12	Increases	(5)
*SGK1*	Increased	+6.94	Increases	(7)
*ABCA1*	Decreased	−6.22	Increases	(27)
*PLA2G1B*	Increased	+6.08	Increases	(4)
*SLC6A4*	Decreased	−5.77	Increases	(37)
*SLC15A2*	Decreased	−5.16	Increases	(5)
*SCN5A*	Decreased	−5.11	Increases	(3)
*LRP1*	Decreased	−5.04	Increases	(3)
*SLC17A8*	Decreased	−5.01	Increases	(5)
*SLC5A3*	Decreased	−4.88	Increases	(3)
*ATP7A*	Decreased	−4.86	Increases	(9)
*SLC39A8*	Decreased	−4.81	Increases	(5)
*XPOT*	Decreased	−4.62	Increases	(2)
*SLC4A1*	Decreased	−4.53	Increases	(13)
*SLCO1A2*	Increased	+4.17	Increases	(19)
*HTT*	Increased	−3.96	Decreases	(25)
*ERBB4*	Decreased	−3.78	Increases	(5)
*SLC7A3*	Decreased	−3.52	Increases	(5)
*SLC7A2*	Decreased	−3.37	Increases	(6)

*FC, Fold change; #, Number in brackets is number of findings that the prediction is based upon*.

##### Generation of heatmaps

**Figure 2:**
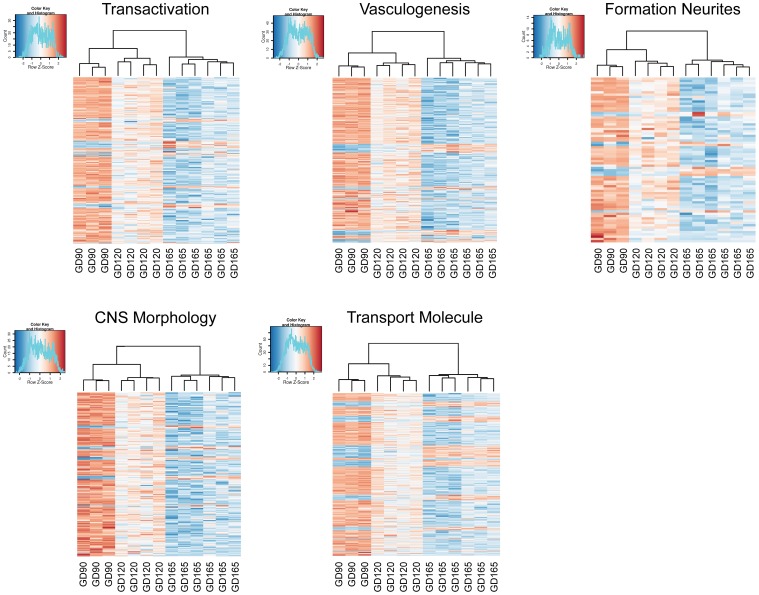
**Heatmaps generated from the read counts for each transcript related to certain gene sets for choroid plexuses at gestation day (GD) 90, GD120 and GD165**. The gene sets were genes annotated to Transactivation, Vasculogenesis, Formation of neurites, Morphology of CNS and Transport of molecule. Note that the cluster analysis shows that animals group into their respective gestational age.

Differentially expressed transcripts/genes, annotated to a certain function, were visualized as heatmaps. Cluster analysis and heatmaps were generated in the statistical software R and presented in Figure 2.

#### Regulatory networks

**Figure 3:**
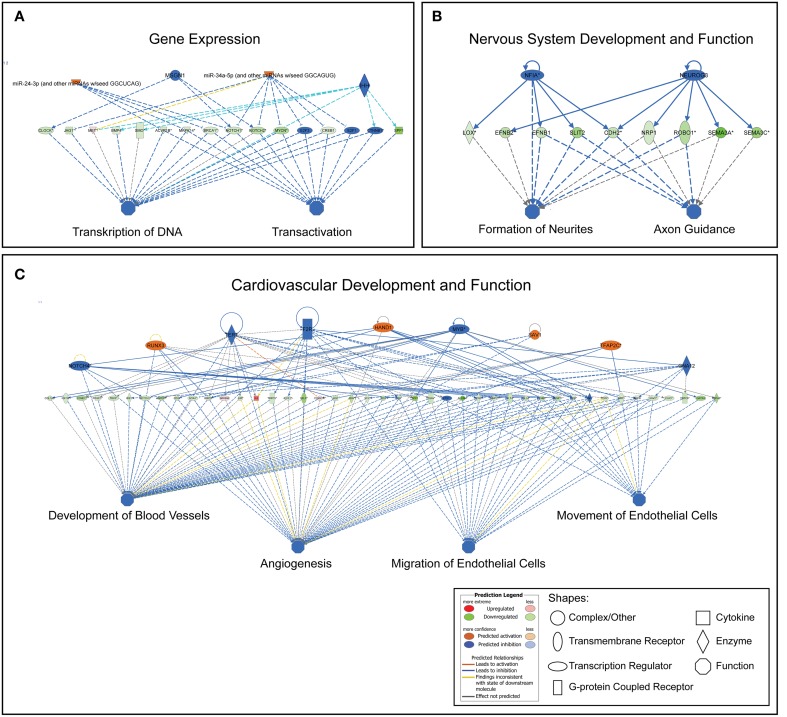
**Most consistent regulatory networks generated for the functions of gene expression (A), nervous system and development (B), cardiovascular system development and function (C)**. This predicts the upstream regulators for the functions tied into the changes in the gene sets. Upstream regulators mainly include transcription factors, enzymes and miRNAs.

Regulatory networks were built from biological functions using transcript profiles from the dataset and required direct connections between molecules based on experimental evidence in the IPA Knowledge Base. The Regulatory networks connect biological functions of regulated genes in the dataset with upstream (top) regulators. We present the most consistent networks relating to the biological functions within Gene Expression, Cardiovascular System Development and Function, Nervous System Development and Function. No extensive and consistent networks could be built from genes categorized to Transport of molecule and none is illustrated. Networks are presented in Figure 3. The top consistent network related to gene expression was associated with transcription of RNA and transactivation (consistency score +6.75) predicting regulating of these functions by an activation of *miRNA-24-3-p*, *miRNA-34a-5p*, while inhibition of *MSGN* and *IHH*. The most consistent network within Cardiovascular functions was related to Blood vessel development, Angiogenesis, and Migration/Movement of endothelial cells (consistency score +11.35). Top regulators of these functions are predicted to be an activation of *RUNX4*, *HAND1*, *SAV1*, *TFAP2C* while inhibition of *NOTCH4*, *TERT*, *F2R*, *MYB*, and *GNA12*. The most consistent network for Nervous System functions was related to Formation of neurites and Guidance of axons (consistency score +3.67) with top regulators of this network being *NFIA* (inhibited) and *NEUROG3* (inhibited).

#### Canonical pathways

Canonical Pathways are well defined pathways involved in intra- and inter-cellular functions. The number of significantly enriched canonical pathways (*p* < 0.01) between GD90 and GD165 was in total 18. For these there is an overrepresentation of genes in our data set for what can be expected from chance. These pathways are presented in Table 7. It should be noted that some of these pathways do have a large overlap of molecules, for instance Axon guidance signaling and Ephrin signaling shares 40 molecules. In addition, we used the Molecular activity predictor in IPA to predict the outcome of changes in these pathways on biological functions.

**Table 7:** **Significantly Enriched canonical pathways (*p* < 0.01) between GD90 and GD165 in the choroid plexus**.

**Canonical pathways**	***p*-value**
Axonal guidance signaling	2.34E-06
Molecular mechanisms of cancer	7.24E-04
Ephrin receptor signaling	7.59E-04
Epithelial adherens junction signaling	9.33E-04
CDK5 signaling	1.17E-03
PTEN signaling	2.09E-03
Protein Kinase A signaling	2.29E-03
Actin cytoskeleton signaling	2.69E-03
Hepatic fibrosis/Hepatic stellate cell activation	3.98E-03
Wnt/β-catenin signaling	4.17E-03
Ephrin A signaling	4.17E-03
Insulin receptor signaling	4.47E-03
Dopamine-DARPP32 feedback in cAMP signaling	7.94E-03
RhoGDI signaling	8.32E-03
Role of NFAT in cardiac hypertrophy	8.51E-03
Sertoli cell-sertoli cell junction signaling	8.91E-03
Gap junction signaling	9.77E-03
GNRH signaling	9.77E-03

## Discussion

The choroid plexus is the site of the blood-CSF barrier. This organ acts like a barrier early in development (Ek et al., 2003; Kratzer et al., 2012; Liddelow et al., 2013). A well-formed barrier is the basis for selective bi-directional transfer of molecules across the plexus. The CSF flow through the ventricular system is often simply thought of as the lymph system of the brain serving as a system for the brain to rid itself of waste products. However, there is now a growing body of evidence that indicating the plexus and its regulation of CSF play an active role in brain development (Falcao et al., 2012; Zappaterra and Lehtinen, 2012). Understanding choroid plexus function during fetal development is central to comprehending normal and abnormal brain development. In order to enhance our understanding of this tissue's normal development we obtained tCP from a primate, the hamadryas baboon, during three different fetal ages, from midterm to late gestation, and sequenced the RNA transcriptome. The strength of this study is that the species studied represents the closest experimental model available to human development and can be obtained in a physiological state at several developmental stages. Post-mortem human tissues are liable to be obtained with questionable quality. Although adult studies have suggested high similarity between the mouse and human choroid plexus transcriptome (Janssen et al., 2013), other studies have revealed mixed overlap of genes across studies of solely the adult mouse choroid plexuses (Marques et al., 2011). These differences may be due to the design of these comparisons. In addition, translation in development from rodents to humans is not straightforward since these two groups of mammals show a different developmental timing of many brain maturation processes. One limitation of the present analysis is that the full baboon genome is not known and we could therefore not fully annotate all transcripts for this primate.

Our analysis reveals that the developmental profile of the plexus transcriptome is age distinct. For every set of transcripts analyzed the three different gestational ages cluster within their age group and with the GD90 and GD120 age groups showing the closest signature of their transcriptome (Figure 2). There are some marked overall developmental changes in the transcriptome. The overall number of transcripts that are age unique in the tCP decreases progressively during fetal development with the greatest number at GD90 and the least at GD165. The exact number is influenced by what is set as “detected” but even when we used different settings for detection level a similar progressive decrease in number of unique transcripts with age was observed, indicating robustness of the analysis. The differential analysis of transcripts also highlights this change in transcriptome in that the majority of significantly changed transcripts were down-regulated both between GD90 and GD120 and between GD120 and GD165; overall from GD90 to GD165 about 2/3 of all significantly changed transcripts were down-regulated. Not only does this observation point to the existence of significant changes in the plexus during development it also suggest that the choroid plexus is more active in younger fetuses than at late gestation with many transcripts only turned on earlier in development. In order to further analyze these changes we used the Ingenuity knowledge base to annotate functions, enrichment of pathways and to predict regulatory networks. The functional annotation suggested very widespread functions across many biological processes during plexus development predicting a decrease in most functions also suggestive of a more active plexus in the younger fetus (see Supplementary Table 1).

In accordance with the decrease in transcripts during development, the functional annotation predicted significant decrease in all biological functions related to gene expression during tCP development with Transactivation showing the most negative activation score (See Table 3A). Table 3B presents the most regulated genes associated with decreased transactivation. Five of these genes have a very strong supporting base for their predicted function and activation state (>30 findings). These are *CREPBP* gene (encoding for cAMP-response element binding protein), *NCA1 and NCA2* gene (encoding for nuclear receptor coactivator 1/2 proteins), *EP300* (encoding for E1A binding protein p300) and *NR3C1* (encoding for the glucocorticoid receptor). The first four of these have histone acytyltransferase activity, which acylates histones making DNA more accessible for transcription and also helps numerous nuclear receptors in transcription. *CREBBP* and its paralog *EP300* can work as epigenetic regulators acetylating key transcription factors such as p53 (Wang et al., 2013). The cytosolic glucocorticoid receptor has many glucocorticoids as ligands and binds to response elements on DNA to turn on genes and has been implicated in many developmental processes.

We found changes similar to those reported in the adult mouse choroid plexus transcriptome in many genes associated to cardiovascular and nervous system development (Marques et al., 2011). In addition, functional annotation showed that Cardiovascular System Development and Function had the second most significant *p*-values (Table 2B). We further explored changes in genes related to the vascular system and in particular development of blood vessels. In general, transcriptional changes predicted a decrease in development of the vascular system during fetal development (Table 4A). In relation to vasculogenesis, it is notable that five of these genes have >20 findings in the knowledge base predicting decrease activation state: *ANGPT1*, encoding for angiopoietin-1 well known to play a role in angiogenesis and stability of blood vessels. *EFNB2* gene, encoding for the ephrin-B2 protein, as well as being involved in angiogenesis it is also known to be involved in the development of the nervous system (Kullander and Klein, 2002). The third is *CDH5*, encoding an endothelial specific cadherin (also known as vascular endothelial cadherin) closely associated to angiogenesis (Daniel and Abrahamson, 2000). The most regulated of the genes associate with inhibition of vasculogenesis was *CDH13*, encoding for cadherin-13 protein (also known as t-cadherin), although studies suggest that this is important for revascularization of tissues (Parker-Duffen et al., 2013), little is known about its role during normal development.

Previous studies of adult choroid plexus transcriptome have shown expression of many genes associated to Nervous System function (Marques et al., 2011; Janssen et al., 2013; Liddelow et al., 2013). In our analysis, the functional annotation showed that Nervous System Development and Function scored one of the highest significant *p*-values for system biology categories (Table 2B). The functional annotation suggests a great variety of effects on the nervous system including neuronal migration, axonal guidance and formation of neurites involving a large number of effector molecules, which cannot all be detailed. However, each individual predicted function only involves quite a small number of proteins (compared to other functions outlined here). Table 5B, listing all changes predicting a decrease in neurite formation, for instance only has nine molecules in total. These changes could be circumstantial, however, interestingly the most enriched pathways in our data set also suggests that there are many gene changes in relation to functions in the nervous system such as axonal guidance signaling, ephrin signaling, CDK5 signaling and dopamine-DARPP32 feedback in cAMP signaling (Table 7). Ephrin and CDK5 signaling pathways overlaid with changes in genes during development are depicted in Supplementary Figures 1, 2. These results do support the notion that signaling systems within the plexus may be regulating some processes during brain development potentially affecting several maturation processes of the brain. There is much evidence that secretory signals from the choroid plexus do affect brain processes both during development and in adult. In the adult it has been shown that neuroblast migration follows the flow of CSF (Sawamoto et al., 2006) and that the choroid plexus can even play a role in plasticity of the visual cortex (Spatazza et al., 2013). Furthermore, fibroblast growth factors, bone morphogenetic proteins, Wnts, and platelet derived growth factors produced by the choroid plexus have been shown to affect brain processes (Falcao et al., 2012). The insulin growth factors in CSF have been shown to be important for brain subventricular zone processes. IGF-1 appears to promote proliferation in the subventricular zone and IGF-2, which is much higher in developing brain CSF, can increase neuronal cell division (Mairet-Coello et al., 2009; Lehtinen et al., 2011). Since the ventricular zone expresses receptors for ephrins it is intriguing that ephrin receptor signaling is one of the most enriched pathways in the development of the primate tCP. It is, however, also possible that these genes and signaling pathways have a specific, as yet unknown role within the plexus itself. There is evidence that axonal guidance molecules are involved in processes in both epithelial as well as endothelial cells (Hinck, 2004).

The choroid plexus contains many transporting systems to exclude or transport molecules into the CNS. There have previously been several studies examining transporters in the plexus in the rodent (Kratzer et al., 2013; Liddelow et al., 2013; Saunder et al., 2013). These studies have shown that many mRNAs for transporters related to protection from xenobiotics are already present in the embryonic rat. In addition, barrier associated genes show little developmental regulation indicating a mature blood-CSF barrier already in the rodent embryo. Although we have not made such a detailed exploration specifically of these genes in the baboon in the present study, we did not obtain any significant z-scores for any transport function of different molecules. In Table 6 we have listed the most regulated genes annotated to Transport of molecules. These are mainly associated to transport of ions, amino acids and neurotransmitters. Notable is that we found a pronounced increase during development in *SLCO1C1* encoding for organic anion transporting polypeptide 1C1 which mediates high affinity transport of thyroxin and reverse-T3 (Pizzagalli et al., 2002). A similar marked increase of *Slco1c1* has been shown to occur between the embryonic to the adult choroid plexus in rats (Kratzer et al., 2013). Since it is known that thyroxin is important for brain development the authors suggested that there must be other transporters mediating sufficient thyroxin to the embryonic brain. Likewise *CP*, encoding for ceruloplasmin, a main carrier of copper/iron ions in the blood, showed a strong increase in development in the baboon. Ceruloplasmin content in choroid plexus has been shown to be very high in the adult and localizes specifically to choroid plexus (Rouault et al., 2009). On the other hand there was a decrease in *ATP7A* and *SLC39A8* transporters transcripts which have affinity for copper/zinc. There was also a decrease in genes encoding for cationic amino acid transporters in the plexus (*SLC7A2, SLC7A3*) as well as proteins associated to neurotransmitter transport (*SLC1A7, SLC6A4, SLC17A8*). This is consistent with previous studies which have showed both higher gene expression of the amino acid solute carriers in the antenatal choroid plexus as well as higher amino acid transport into the fetal brain as compared to the adult (Saunder et al., 2013).

Functional annotation to Cellular Organization and Assembly function had the most significant *p*-value of all functions and Organization of cytoskeleton one of the most negative activation scores (−7.02; Supplementary Table 1). In addition, several of the enriched canonical pathways are related to cellular assembly and particular actin cytoskeletal processes such as the actin cytoskeletal signaling, RhoGI signaling, gap junction signaling, and GNRH signaling pathway. We further explored the gene changes within these pathways using molecular activity predictions in IPA, which showed that these pathways predicted an increase in actin stabilization while a decrease in actin reorganization with development (see Supplementary Figure 3 for example of molecular predictions). Taken together this suggests that many of the gene changes in the tCP during development are linked to cytoskeletal processes and strengthening the cytoskeleton appears to be one such cellular process as the epithelium matures.

In conclusion, to our knowledge this is the first analysis of development of the fetal non-human primate choroid plexus transcriptome. Our data point to a more active choroid plexus earlier in fetal development with regulation of a more unique set of genes. We report progressive distinct transcriptome changes with widespread changes to genes across a great range of biological functions. Many of these functional changes are those that could be predicted on the basis of known changes during plexus development. The extent of the changes and nature of the genes involved indicate that the developing plexus is undertaking specific active functions with precise regulation and not just acting as a developmental barrier. Importantly, our observations support the notion that the choroid plexus has a direct, active role in influencing brain development. Future studies should further examine the role of these pathways in the choroid plexus.

### Conflict of interest statement

The authors declare that the research was conducted in the absence of any commercial or financial relationships that could be construed as a potential conflict of interest.

## Supplementary material

Supplementary Figure 1 **Diagrams of canonical ephrin signaling pathway showing up (red) and down (green) regulated genes between GD90 and GD165 along with predictions on biological functions**.

Supplementary Figure 2 **Diagrams of canonical CDK5 signaling pathway showing up (red) and down (green) regulated genes between GD90 and GD165 along with predictions on biological functions**.

Supplementary Figure 3 **Example of molecular activity prediction using Ingenuity Pathway Analysis software for canonical Actin signaling pathway from differentially expressed genes between GD90 and GD165**. This connects the gene changes in the actin signaling pathway to biological functions. The changes to this pathway predict an increase in actin polymerization and actin stabilization whereas actin reorganization is decreased.

Supplementary Table 1 **Function annotated genes to all subcategory functions with a significant activation z-score (>+2 or <−2)**. #, Number of annotated genes to function.
