# Supplementary figures and images for: Transcriptomal changes and functional annotation of the developing non-human primate choroid plexus
Source: Front Neurosci. 2015 Mar 12;9:82. doi: 10.3389/fnins.2015.00082 (PMC4357249; doi:10.3389/fnins.2015.00082)

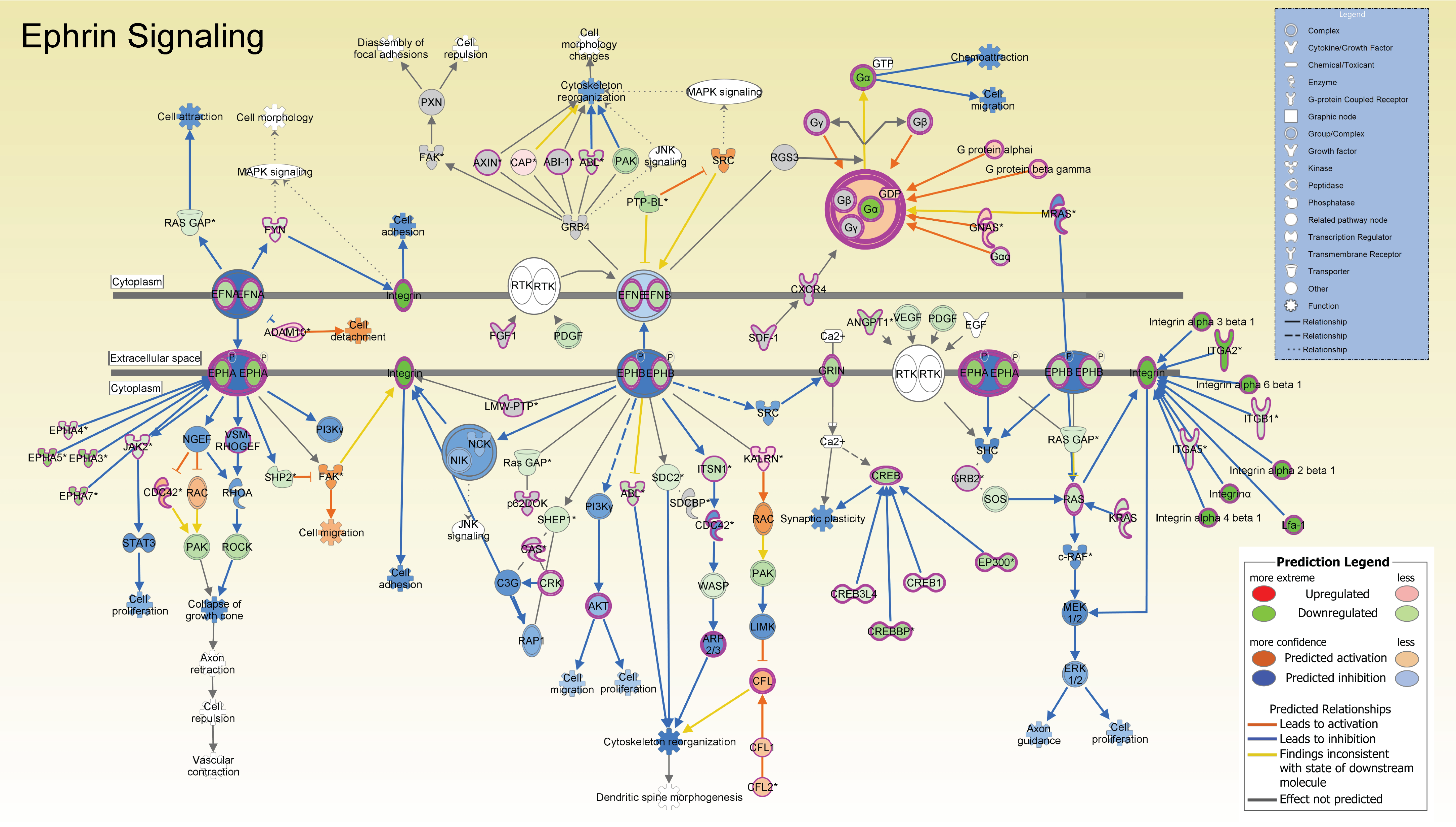

Supplement: Supplementary Figure 1 — Diagrams of canonical ephrin signaling pathway showing up (red) and down (green) regulated genes between GD90 and GD165 along with predictions on biological functions. [file Image1.TIF]

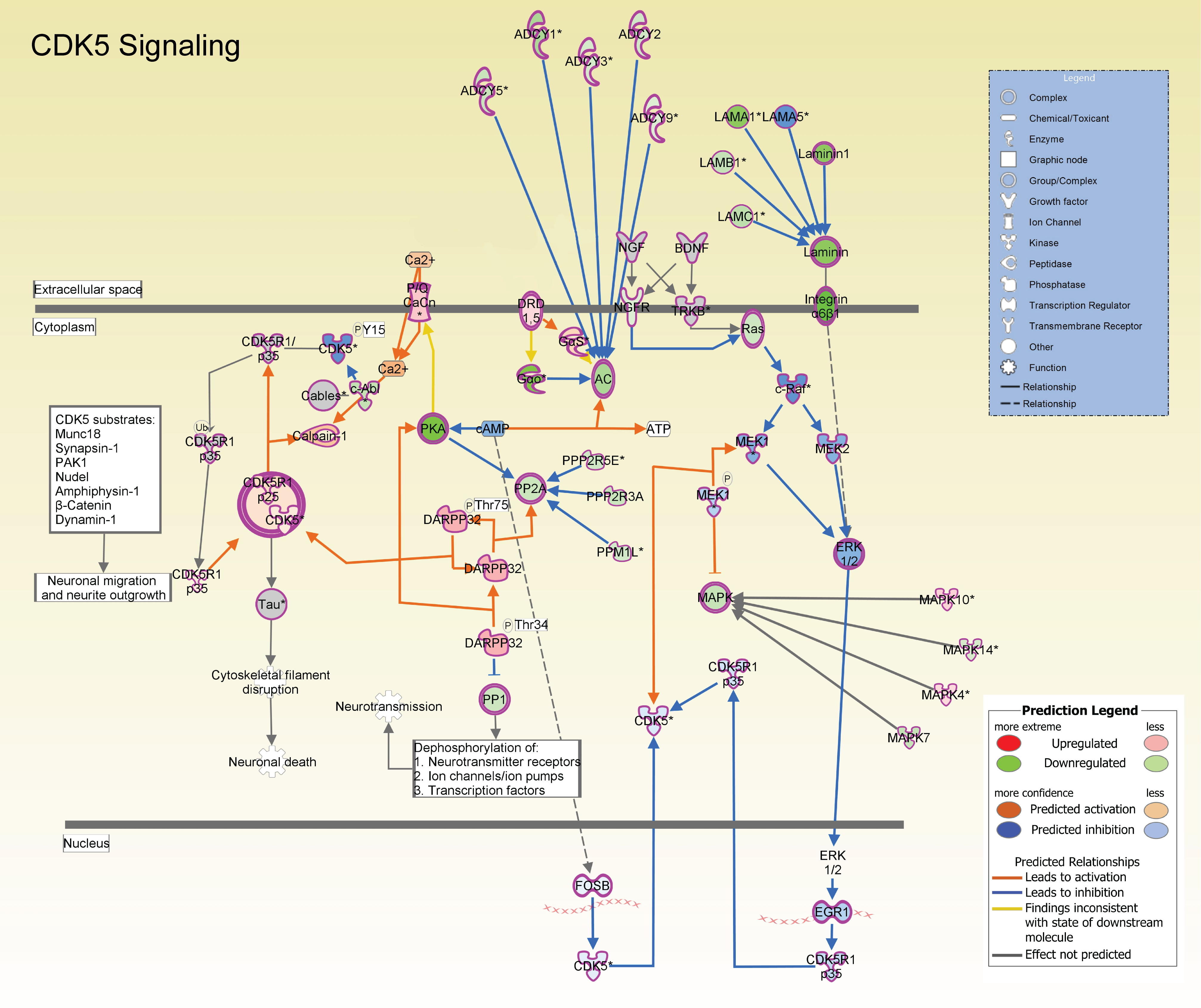

Supplement: Supplementary Figure 2 — Diagrams of canonical CDK5 signaling pathway showing up (red) and down (green) regulated genes between GD90 and GD165 along with predictions on biological functions. [file Image2.TIF]

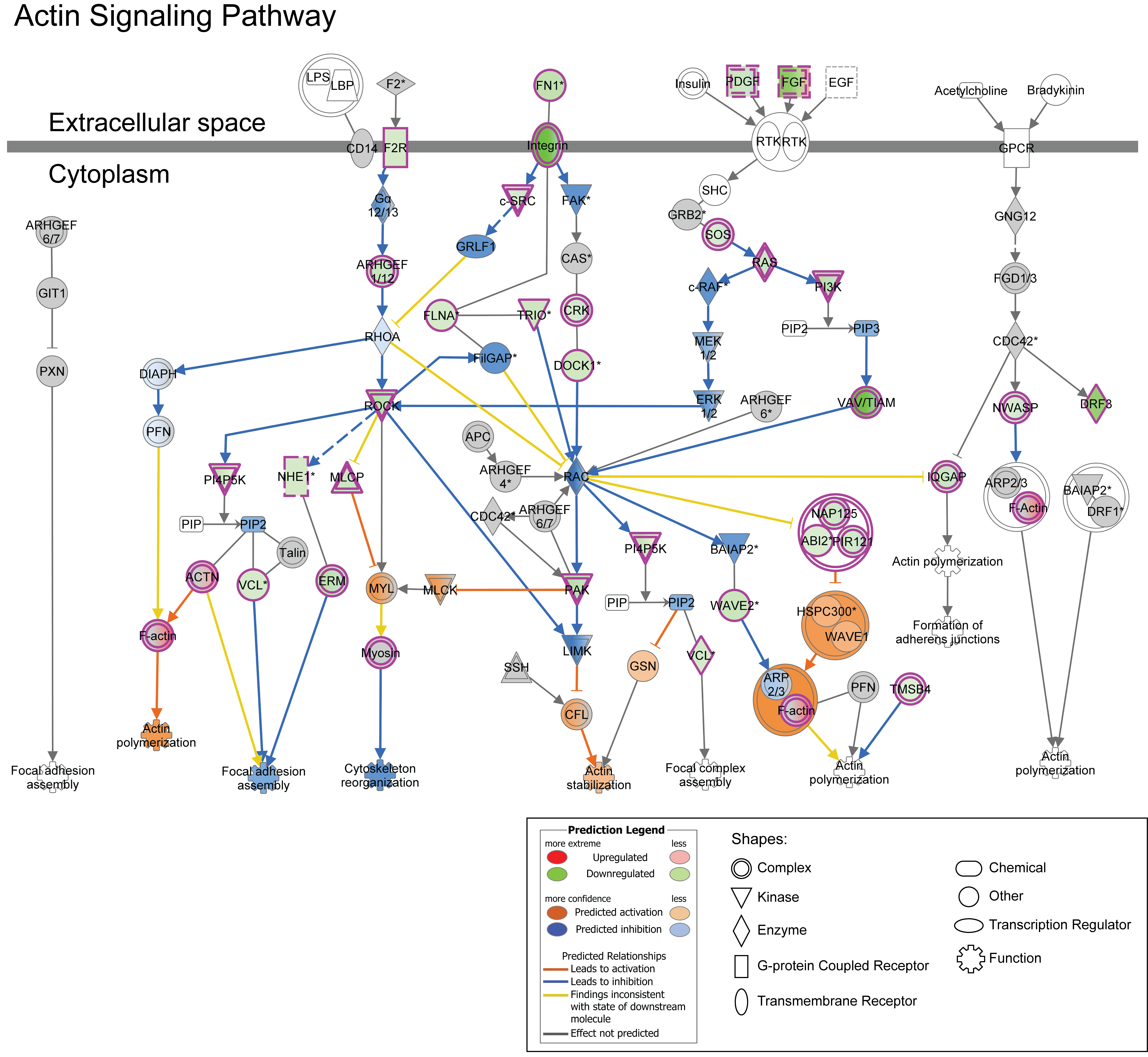

Supplement: Supplementary Figure 3 — Example of molecular activity prediction using Ingenuity Pathway Analysis software for canonical Actin signaling pathway from differentially expressed genes between GD90 and GD165. This connects the gene changes in the actin signaling pathway to biological functions. The changes to this pathway predict an increase in actin polymerization and actin stabilization whereas actin reorganization is decreased. [file Image3.TIF]
