# Supplementary material for: Transcriptomal changes and functional annotation of the developing non-human primate choroid plexus
Source: Front Neurosci. 2015 Mar 12;9:82. doi: 10.3389/fnins.2015.00082 (PMC4357249; doi:10.3389/fnins.2015.00082)
Supplement: Supplementary Table 1 — Function annotated genes to all subcategory functions with a significant activation z-score (>+2 or <−2). #, Number of annotated genes to function. [file Table1.DOCX]

| Functions Annotation | z-score | # |
| --- | --- | --- |
| adhesion of connective tissue cells | -2.32 | 50 |
| aggregation of cells | -3.13 | 86 |
| angiogenesis | -2.39 | 234 |
| apoptosis | +3.50 | 650 |
| apoptosis of epithelial cell lines | -3.81 | 61 |
| apoptosis of neurons | +3.08 | 110 |
| branching of cells | -2.40 | 127 |
| branching of epithelial tissue | -2.29 | 38 |
| cell death | +4.18 | 824 |
| cell death of epithelial cell lines | -3.48 | 77 |
| cell movement | -4.87 | 570 |
| cell movement of brain cells | -2.96 | 35 |
| cell movement of central nervous system cells | -2.38 | 39 |
| cell movement of endothelial cells | -2.38 | 111 |
| cell movement of neurons | -3.03 | 93 |
| cell spreading | -4.36 | 82 |
| cell survival | -7.07 | 361 |
| cell viability | -7.02 | 326 |
| closure of embryonic tissue | -2.89 | 40 |
| closure of neural tube | -2.14 | 37 |
| development of abdomen | -3.11 | 134 |
| development of blood vessel | -2.46 | 268 |
| development of body trunk | -4.14 | 287 |
| development of brain | -2.01 | 157 |
| development of cardiovascular system | -2.58 | 328 |
| development of cytoplasm | -2.26 | 128 |
| development of extraembryonic tissue | -2.62 | 30 |
| expression of DNA | -3.36 | 337 |
| expression of RNA | -4.15 | 464 |
| extension of cellular protrusions | -2.30 | 73 |
| extension of neurites | -2.21 | 58 |
| extension of plasma membrane projections | -2.20 | 62 |
| fibrogenesis | -2.78 | 122 |
| formation of blood vessel | -2.15 | 33 |
| formation of cells | -3.57 | 126 |
| formation of cellular protrusions | -5.74 | 266 |
| formation of cytoskeleton | -2.42 | 111 |
| formation of filaments | -3.03 | 119 |
| formation of neurites | -3.23 | 59 |
| formation of plasma membrane | -2.77 | 71 |
| formation of plasma membrane projections | -2.95 | 185 |
| growth of connective tissue | -2.76 | 173 |
| growth of embryo | -2.79 | 118 |
| growth of organism | -3.33 | 209 |
| guidance of axons | -2.59 | 55 |
| interphase | -2.88 | 160 |
| invasion of cells | -4.92 | 240 |
| length of animal | -8.73 | 252 |
| long-term potentiation | -3.87 | 77 |
| long-term potentiation of brain | -2.05 | 43 |
| long-term potentiation of cerebral cortex | -2.38 | 39 |
| long-term potentiation of hippocampus | -2.36 | 38 |
| microtubule dynamics | -6.82 | 357 |
| migration of cells | -4.59 | 515 |
| migration of endothelial cells | -2.57 | 97 |
| migration of neurons | -2.66 | 89 |
| migration of vascular endothelial cells | -2.82 | 52 |
| morphogenesis of cardiovascular system | -3.08 | 72 |
| morphogenesis of epithelial tissue | -2.25 | 41 |
| morphology of body cavity | +3.01 | 308 |
| morphology of brain | +4.06 | 148 |
| morphology of cardiovascular system | +3.06 | 224 |
| morphology of cells | +2.78 | 549 |
| morphology of central nervous system | +4.06 | 162 |
| morphology of connective tissue | +2.16 | 129 |
| morphology of extraembryonic tissue | +2.43 | 71 |
| morphology of forebrain | +3.13 | 75 |
| morphology of head | +5.97 | 267 |
| morphology of nervous system | +4.88 | 257 |
| morphology of telencephalon | +3.13 | 60 |
| movement of vascular endothelial cells | -2.76 | 53 |
| necrosis | +2.44 | 650 |
| neonatal death | +8.20 | 136 |
| organismal death | +16.05 | 660 |
| organization of actin cytoskeleton | -2.63 | 81 |
| organization of cytoplasm | -7.08 | 441 |
| organization of cytoskeleton | -7.02 | 422 |
| outgrowth of cells | -2.50 | 140 |
| perinatal death | +9.97 | 192 |
| phosphorylation of L-amino acid | -3.02 | 62 |
| phosphorylation of L-tyrosine | -3.15 | 47 |
| phosphorylation of protein | -2.46 | 207 |
| plasticity of synapse | -2.73 | 36 |
| proliferation of cells | -6.36 | 935 |
| proliferation of connective tissue cells | -2.90 | 164 |
| proliferation of fibroblast cell lines | -2.13 | 109 |
| proliferation of fibroblasts | -3.27 | 93 |
| proliferation of neuronal cells | -2.75 | 174 |
| quantity of cells | -4.91 | 416 |
| quantity of cellular protrusions | -2.77 | 49 |
| S phase of connective tissue cells | -3.38 | 18 |
| S phase of fibroblasts | -3.23 | 17 |
| size of body | -8.77 | 251 |
| size of brain | -2.81 | 31 |
| sprouting | -2.66 | 129 |
| synthesis of DNA | -2.03 | 115 |
| transactivation | -5.22 | 149 |
| transcription | -4.01 | 428 |
| transcription of DNA | -2.84 | 319 |
| transcription of RNA | -3.80 | 419 |
| vasculogenesis | -3.15 | 245 |
